# A 2 Year Audit Cycle Monitoring Cardiovascular Risk Factors in Patients Taking Atypical Antipsychotics

**DOI:** 10.1192/bjo.2026.11705

**Published:** 2026-06-30

**Authors:** Kristin Hawkins

**Affiliations:** Warwick Medical School, Coventry, United Kingdom

## Abstract

**Aims:**

Atypical antipsychotics are a class of medication used to treat psychiatric disorders involving psychosis. Alongside other side effects, they are strongly associated with increase the risk of adverse cardiovascular outcomes e.g. hyperlipidaemia and weight gain. As such, the BNF states that patients should be monitored annually for these risk factors, including weight, glucose/Hbs-648c, blood lipids and blood pressure.

**Aims:**

To assess the monitoring of cardiovascular risk factors for vulnerable patients on atypical antipsychotics at a general practice in the West Midlands to allow interventions and modifications to be put in place. In the second cycle, the aim was to assess whether interventions and recommendations made from the first audit made a significant difference to levels of monitoring.

**Methods:**

Searches on EMIS were conducted from a list of registered patients currently on atypical antipsychotics to assess whether weight, glucose/HbA1c, blood lipids and BP had been checked in the previous year, in accordance with BNF recommendations for monitoring. Standards set were:

- **100% of patients will have three or more of the four CVD risk factors monitored within the past year.**
- **80% of patients in the practice monitored for each measure within the past year.**
- **A statistically significant increase in overall rates of monitoring from 2024 to 2025 using Fisher’s Exact Test.**

**Results:**

For standard 1, there was an increase from 48% to 60% of patients who met this standard from 2024 to 2025. For standard 2, none of the four measures met the criteria in either year, however three measures had increased from 2024. Standard 3 was met as the overall monitoring rate had increased from 58% in 2024 to 65% in 2025, which was a statistically significant increase using Fisher’s Exact test with a p value of 0.04.

**Conclusion:**

NICE guidelines state that patients taking antipsychotics should have annual monitoring of cardiovascular risk factors due to their potential side effects. Although results show that neither of the first two standards were met in both cycles, standard 3 was met, showing a statistically significant improvement in rates of monitoring. This suggested that recommendations made from the 2024 cycle had a positive effect on rates of cardiovascular monitoring of patients on antipsychotics but that further improvements could be made. Further recommendations were made, including implementing manual monitoring reminders for patients not under the SMI checks and long-term solutions for phlebotomy access. Another re-audit will be conducted to assess the impact of these recommendations.

